# Edge states and integer quantum Hall effect in topological insulator thin films

**DOI:** 10.1038/srep13277

**Published:** 2015-08-25

**Authors:** Song-Bo Zhang, Hai-Zhou Lu, Shun-Qing Shen

**Affiliations:** 1 Department of Physics, The University of Hong Kong, Pokfulam Road, Hong Kong, China

## Abstract

The integer quantum Hall effect is a topological state of quantum matter in two dimensions, and has recently been observed in three-dimensional topological insulator thin films. Here we study the Landau levels and edge states of surface Dirac fermions in topological insulators under strong magnetic field. We examine the formation of the quantum plateaux of the Hall conductance and find two different patterns, in one pattern the filling number covers all integers while only odd integers in the other. We focus on the quantum plateau closest to zero energy and demonstrate the breakdown of the quantum spin Hall effect resulting from structure inversion asymmetry. The phase diagrams of the quantum Hall states are presented as functions of magnetic field, gate voltage and chemical potential. This work establishes an intuitive picture of the edge states to understand the integer quantum Hall effect for Dirac electrons in topological insulator thin films.

The discovery of the integer quantum Hall effect in two-dimensional electron gas opens a window to explore topological phases in quantum matter^1^,^2^. In the quantum Hall effect the longitudinal conductance vanishes while the Hall conductance *σ*_*xy*_ is quantized at *ve*^2^/*h*, where *e* is the elementary charge and *h* is the Planck constant. It is known that the integer *v* is the topological invariant of a quantum phase, it counts the number of conducting chiral channels at the edges of the system, and is insensitive to the geometry of the sample, impurity, and interactions of electrons^3^,^4^,^5^,^6^. Three-dimensional topological insulator is a new class of topological materials and is characterized by the formation of the Dirac fermion gas covering its surfaces^7^,^8^,^9^,^10^. Soon after the discovery of three-dimensional topological insulators, the formation of the Landau level (LL) of the surface Dirac electrons in a strong magnetic field has been observed by the scanning tunneling microscope^11^,^12^,^13^ and Shubnikov-de Haas oscillations in the longitudinal conductance^14^,^15^,^16^. It has been known since 1980’s that the Hall conductance of massless Dirac fermions is quantized as half integers *v* = *n* + 1/2, where *n* = 0, ±1, ±2,….^17^,^18^,^19^,^20^,^21^, and 1/2 is attributed to the Berry phase of the massless Dirac fermions acquiring from a cyclotron motion around the Fermi surface^19^,^22^,^23^. Usually each LL carries one conducting channel near the edge due to the geometric distortion of the cyclotron motion of electrons in a magnetic field. The quantum Hall conductance is determined by the number of the edge states^4^,^5^. Thus the relation between the half-quantized Hall conductance and the number of the conducting edge channel is an open issue and has attracted a lot of studies^20^,^21^,^24^,^25^,^26^,^27^,^28^,^29^,^30^,^31^,^32^,^33^,^34^,^35^,^36^. Very recently the integer quantum Hall effect has been measured in three-dimensional topological insulator thin films by two independent groups^37^,^38^. One group measured a series of plateaux of *v* = −1, 0, 1, 2, 3 and *v* = 1, 3 in BiSbTeSe_2_^37^, and the other measured the plateaux of *v* = 0, ±1 in (Bi_1−*x*_Sb_*x*_)_2_Te_3_^38^. These plateaux of the Hall conductance are attributed to the addition of the top and bottom surface electrons, and always yield integers in units of *e*^2^/*h*.

In this work we present solutions of the LLs and edge states of the surface electrons, and explore the formation of the quantum Hall effect in a topological insulator thin film. The Hall conductance is calculated by means of the Kubo formula at zero temperature, which is well quantized when the chemical potential lies between two LLs. Two distinct patterns of the quantum Hall conductance are found: *v* is an odd integer when the LLs of the top and bottom surface electrons are degenerate in a thick film, and *v* is an integer when structure inversion symmetry is broken in the film or the top and bottom surface electrons are coupled to open an energy gap due to the finite-size effect^39^,^40^. The absence of the *v* = 0 plateau is caused by the degeneracy of two sets of LLs, and the *v* = 0 plateau emerges when the degeneracy is lifted by the finite-size effect or structure inversion asymmetry (SIA, which can be produced by the energy difference between the two surfaces). The width of the *v* = 0 plateau is determined by the energy difference between the two LLs closest to zero energy. The state of *v* = 0 can be either in the quantum spin Hall phase or trivial band insulator phase, and the quantum spin Hall effect can be easily broken down by SIA.

## Results

### Model

**Figure 1:**
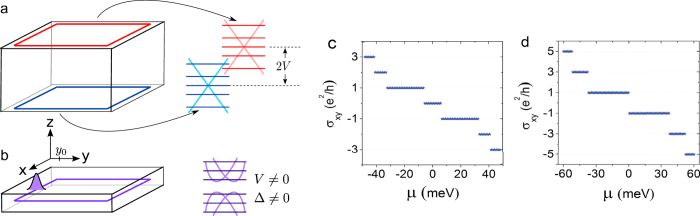
Two distinct quantum Hall conductance patterns. (**a**) Schematics of LLs of the surface states in a thick topological insulator film under a perpendicular magnetic field. Each of the top and bottom surfaces hosts an independent set of LLs. 2*V* is the energy offset between the top and bottom surfaces, usually induced by SIA and tunable with a gate voltage. (**b**) The same as (**a**) except for a thin film, where the LLs reside in the whole film rather than at specific surfaces. Δ is the finite-size gap opened by the hybridization of the top and bottom surface states. (**c**) The pattern of integer quantum Hall conductance plateaux as a function of the chemical potential *μ* in a fixed magnetic field *μ*_0_*H* for a general case. (**d**) The pattern of odd integer quantum Hall plateaux in the case where the two sets of LLs of the top and bottom surface states are degenerate.

Consider a thin film of three-dimensional topological insulator. Starting from the bulk Hamiltonian for topological insulators^10^,^41^, the low-energy effective Hamiltonian for the surface electrons has been derived by solving the differential equation for the bulk bands exactly^39^,^40^


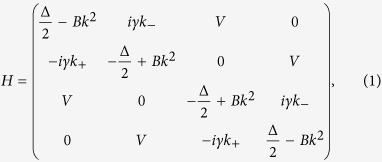


where *k*_±_ = *k*_*x*_ ± *ik*_*y*_ and 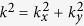 with *k*_*x*,*y*_ the wave vector in the surface plane. The mass term Δ/2 − *Bk*^2^ is generated by the hybridization between the wave functions of the top and bottom surface electrons. Both *B* and Δ depend on the film thickness and approach zero simultaneously in a thick film. 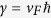 with *v*_*F*_ the effective velocity and 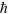 the reduced Planck constant. 2*V* is the potential difference between the top and bottom surfaces due to SIA as shown in Fig. 1a, which can be induced in a realistic thin film by the potential difference between the substrate and vacuum surfaces, and tunable by a gate voltage. The interplay of SIA and top-bottom hybridization gives the Rashba-like splitting in the band structure (see Fig. 1b). The physics of this model has been confirmed by the angle-resolved photoemission spectroscopy experiments on topological insulator thin films^42^,^43^.

### Two patterns of quantum Hall plateaux

For a thin film of topological insulator in the presence of a perpendicular magnetic field, the Hall conductance is usually quantized as an integer *v* 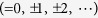 as shown in Fig. 1c, or can only be an odd integer *v* 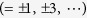 when the LLs of the top and bottom surface electrons coincide exactly as shown in Fig. 1d. Consider a topological insulator thin film. The surface states cover the top and bottom surfaces as illustrated in Fig. 1a,b. The lateral side is also covered by the surface electrons, but is ignored in the present work. For a film with a relatively large thickness, the top and bottom surface states are well separated. Thus the system consists of two decoupled massless Dirac electrons. The two Dirac points can be separated by the potential difference 2*V* between the top and bottom surfaces. The value of *V* then can determine the quantized pattern of the Hall conductance such as the width of quantum plateaux and the existence of *v* = 0 plateau. For *V* *=* 0, the LLs of the top and bottom surface electrons are degenerate, and the Hall conductance is quantized as odd integers *v* = 2*n* + 1, which can be regarded as the addition of two sets of half-quantized Hall conductance, i.e., 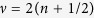. This is very similar to that in graphene, in which the Hall conductance is 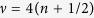, where the factor 4 is attributed to the spin and valley degrees of freedom^44^,^45^. For 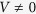, the degeneracy of the LLs is removed, and the Hall conductance becomes quantized as integers in Fig. 1c. Another factor leading to the lift of the degeneracy is the finite-size effect. When the thickness of a thin film is comparable with the spatial distribution of the wave functions of the surface states, the overlap of the wave functions will open an energy gap Δ at the Dirac points of the two surface states^39^, leading to the presence of the *v* = 0 plateau and a possible topological phase of the quantum spin Hall effect.

### Landau levels and edge states

**Figure 2:**
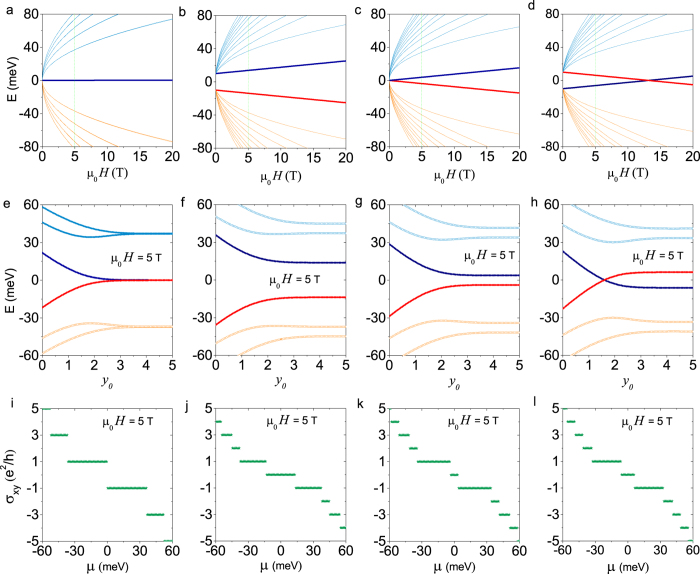
Landau levels and edge states in the absence of SIA. The four columns compare cases with different Δ and B, two parameters in the mass term of the model. From left to right, (i) Δ = 0 and *B* → 0; (ii) Δ*B* < 0; (iii) Δ = 0 and *B* ≠ 0; (iv) Δ*B* > 0. The top row is for the fan diagrams, i.e., the energies of LLs as functions of the magnetic field *μ*_0_*H*. The calculation of the Landau level spectra assumes no boundary. The two LLs of *n* = 0 are highlighted. The middle row is for the energy dispersions of LLs at *μ*_0_*H* = 5 T near an open edge at *y* = 0. y_0_ is the position of guiding center in units of magnetic length 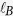. The bottom row is for the Hall conductance *σ*_*xy*_ as a function of the chemical potential *μ* at *μ*_0_*H* = 5 T. For Δ*B* > 0, the two LLs of *n* = 0 cross at a critical magnetic field in (**d**) showing a field-induced quantum phase transition from the quantum spin Hall phase in weak field to trivial band insulator phase in strong field; correspondingly in (**h**) the higher hole-like LL and the lower electron-like LL of *n* = 0 cross when approaching the edge, contributing to two conducting channels with opposite velocities when the chemical potential crosses them. This characterizes the quantum spin Hall phase with no charge Hall conductance but a finite quantized spin Hall conductance. For both cases (ii) and (iii), all electron-like LLs are above all hole-like LLs, therefore there is no quantum spin Hall phase; For case (i) of Δ = 0 and *B* → 0, all LLs in (**a**) are two-fold degenerate in the bulk, but the degeneracy is lifted in (**e**) when approaching the edge. In all cases *B* = −500 meVnm^2^ and *γ* = 300 meVnm for comparison, while Δ = 0 in cases (i) and (iii), 20 meV in case (ii), and −20 meV in case (iv).

**Figure 3:**
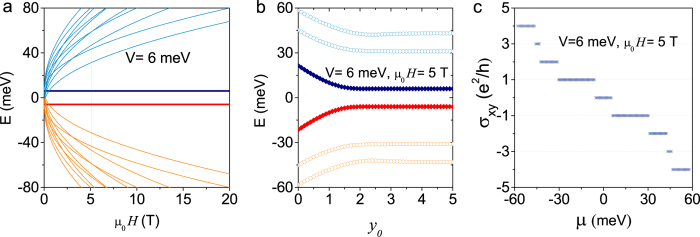
Integer quantized Hall plateaux due to SIA. For case (i) with Δ = 0 and *B* → 0 but a finite *V* = 6 meV, (**a**) the fan diagram, (**b**) the energies of the two LLs of *n* = 0 near the edge, and (**c**) the Hall conductance as a function of the chemical potential *μ*. SIA breaks the degeneracies of all LLs in the thick film where Δ = 0 and *B* → 0. As a result, even integer Hall conductance plateaux also appear.

In Figs 2 and 3, we present the LLs in a magnetic field *μ*_0_*H* normal to the thin film, the LL energies or edge states near one edge of the system, and the corresponding patterns of the quantum Hall conductance. In the absence of SIA, i.e., *V* = 0, four possible typical cases are shown in Fig. 2. Case (i) is for a thick film, i.e., Δ = *B* = 0. In the bulk, the LLs of zero energy are degenerate, and are insensitive to the field, as shown in Fig. 2a. However they split into two branches when approaching one edge: one branch goes upward (called electron-like) and the other goes downward (called hole-like), as shown in Fig. 2e. The position 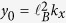 is the guiding center of the wave packages of surface LLs and is proportional to the wave vector *k*_*x*_, where the magnetic length 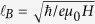. The slope of the energy dispersion near the edge 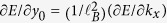 is proportional to the effective velocity of the edge states 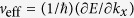, which defines the current flow of the edge states. The Hall conductance is equal to *v* = 1 when the chemical potential is below the LLs of *n* = 0 and *v* = −1 when the chemical potential is above the LLs of *n* = 0. The plateau of *v* = 0 is absent and other plateaux are 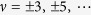. For a thinner film, there exist three cases, (ii)Δ > 0, (iii) Δ = 0, and (iv) Δ < 0, all with the parameter *B* < 0 (without loss of generality we assume negative *B*). In case (ii) with Δ*B* < 0, the two LLs of *n* = 0 are separated in a finite field as shown in Fig. 2b. When approaching the edge the LLs with positive energies go upward while the LLs with negative energies go downward, indicating that the edge electrons with opposite energies move in opposite directions. One of the key features of the quantum Hall conductance is the emergence of the *v* = 0 plateau, and the width of the plateau is determined by the energy difference between the two LLs of *n* = 0 (see Fig. 2j). In case (iii) with Δ = 0 and *B* < 0, the two LLs near the Dirac point are degenerate at zero field, as shown in Fig. 2c. The degeneracy is lifted by a finite field *μ*_0_*H*. The energies of edge states (see Fig. 2g) and the Hall conductance (see Fig. 2k) are very similar to those in case (ii). In case (iv) with Δ*B* > 0, two LLs near zero energy are separated in weak fields, and cross at a finite field as shown in Fig. 2d, indicating a quantum phase transition. In a weak field, the pattern of the Hall conductance (see Fig. 2l) is also similar to that of case (ii), while the dispersions of the edge states (see Fig. 2h) are different. The two LLs of *n* = 0 cross near the edge, which is a key feature of the quantum spin Hall effect in a finite field^46^. At magnetic fields higher than the energy crossing in Fig. 2d, the LLs near zero energy never cross near the edge, similar to those in Fig. 2 f and g, indicating the breakdown of the quantum spin Hall effect in a magnetic field. However, the plateau of *v* = 0 still survives.

In the presence of SIA, 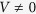, the relative positions of two sets of LLs from the top and bottom surfaces can be tuned by the value of *V*. A typical pattern of the quantum Hall conductance is presented in Fig. 3. The Hall conductance covers all integers. In the case that the chemical potential crosses some accidental crossing points of two LLs, the Hall conductance can change by 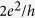.

### Quantum spin Hall state and its breakdown

**Figure 4:**
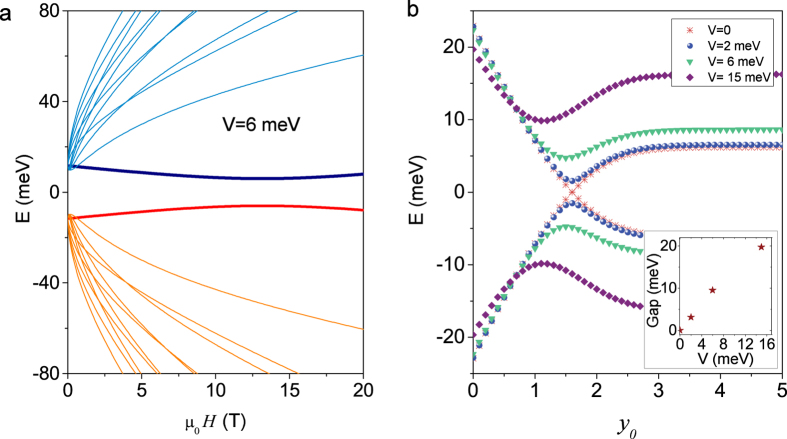
SIA-induced breakdown of the quantum spin Hall phase. (**a**) The fan diagram in the presence of SIA, i.e., *V* ≠ 0. SIA turns the crossing between the two LLs of *n* = 0 in Fig. 2d into an anti-crossing. (**b**) The energies of the two LLs of *n* = 0 at 5 T near the edge for different *V*. In the presence of SIA, the two LLs do not cross near the edge and open an energy gap. Inset: the gap opened between the two LLs of *n* = 0 as a function of *V*. The parameters are *γ* = 300 meVnm, Δ = −20 meV, and *B* = −500 meVnm^2^.

A question arises from the appearance of the quantum spin Hall effect in case (iv) with Δ*B* > 0. Usually the quantum spin Hall effect is protected by time reversal symmetry^47^,^48^. Applying a magnetic field breaks time reversal symmetry, but it is known from the calculation of the Hall conductance that the quantum spin Hall effect can be stabilized in a finite field when the system possesses an intrinsic and hidden s_*z*_ symmetry which guarantees the decoupling of the two blocks with spin up and down in the Hamiltonian^49^. In the presence of SIA, 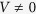, the situation changes. The energy crossing of the two *n* = 0 LLs in a finite field in Fig. 2d becomes an anti-crossing for a finite *V* in Fig. 4a, showing that there is no field-induced quantum phase transition from the quantum spin Hall insulator to the trivial band insulator in the presence of SIA and the system remains in the trivial phase at all magnetic fields. In other words, the quantum spin Hall effect breaks down as long as SIA is present. The breakdown of the quantum spin Hall phase can also be seen from the energies of the edge states in Fig. 4b, in which the energies of the *n* = 0 LLs cross for *V* = 0 while open a finite gap for a nonzero *V*. The gap increases with increasing *V*. For a small *V*, the corresponding energy gap of the edge states is also small. Although the edge states are no longer protected topologically, it can still produce some physical phenomena such as the spin accumulation, or the intrinsic spin Hall effect near the edge when an electric current is applied. However these effects will be weakened for a large *V*. Therefore, the quantum spin Hall effect does not survive in the presence of both a magnetic field and SIA.

### Phase diagrams

**Figure 5:**
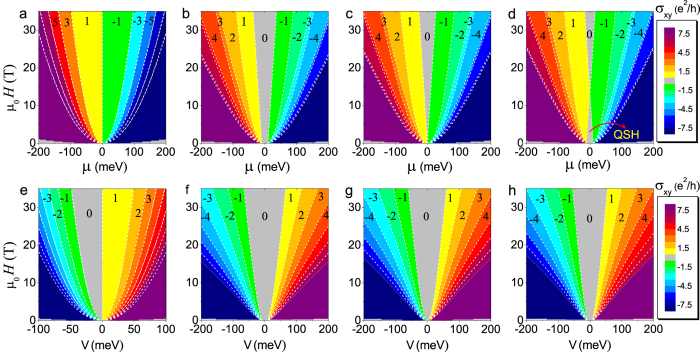
Phase diagrams of the quantum Hall effect in topological insulator films. (**a**–**d**) The phase diagrams as functions of the chemical potential *μ* and magnetic field *μ*_0_*H* in the absence of SIA, i.e., *V* = 0. Different phases are denoted by corresponding Hall conductance *σ*_*xy*_ in units of *e*^2^/*h*. The white dotted lines are the boundaries between different phases. The four columns compare cases with different finite size gap Δ and *B*. From left to right, (i) Δ = 0 and *B* → 0; (ii) Δ*B* < 0; (iii) Δ = 0 and *B* ≠ 0; (iv) Δ*B* > 0. The parameters for different cases are the same as those in Fig. 2. *σ*_*xy*_ is antisymmetric with respect to *μ*. In (**a**) there are only odd integer quantum Hall phases. In (**b**–**d**) there are both odd and even integer quantum Hall phases. In (**d**) the quantum spin Hall (QSH) phase is marked. (**e**–**h**) The phase diagrams as functions of *V* and *μ*_0_*H* while fixing the chemical potential *μ* = −V − 0^+^. Both odd and even integer quantum Hall phases can be induced by changing *V* in all the four cases.

In the absence of SIA, i.e., *V* = 0, we plot in Fig. 5a–d the corresponding phases diagrams of the Hall conductance as a function of the chemical potential *μ* and magnetic field *μ*_0_*H* for the four cases. Different quantum Hall phases are denoted by the quantized Hall conductances and are separated by the boundaries (marked by the white dotted lines). The Hall conductance is antisymmetric with respect to *μ*. In case (i), there are only odd integer quantum Hall phases (see Fig. 5a). The spacings of the phases grow with increasing magnetic field or 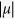. Thus the quantum Hall plateaux are more visible by varying *μ* in a stronger magnetic field, or by varying the magnetic field with a larger fixed 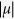. In contrast, not only odd but also even integer quantum Hall phases are possible in the presence of a finite mass, i.e., 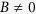 or 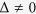 (see Fig. 5b–d). When *μ* = 0, all negative LLs are filled and all positive LLs are empty no matter how large the magnetic field is, and the corresponding Hall conductance is zero, regardless of whether there is energy crossing near the edge of the system. The quantum spin Hall effect only appears in the case of Δ*B* > 0 and *V* = 0 in a weak field, and disappears in a stronger field as shown in Fig. 5d.

Moreover, we calculate the Hall conductance as a function of *V* and magnetic field *μ*_0_*H* in Fig. 5e–h, where we set *μ* = −*V* − 0^+^. An infinitesimal value 0^+^ is introduced to avoid the alignment of the chemical potential *μ* with one of the *n* = 0 LLs (*E*_0_ = −*V*) in case (i) in Fig. 5e. The asymmetry of the Hall conductance with respect to *V* reflects the fact that both of the *n* = 0 LLs (*E*_0_ = ±*V*) are empty for a positive *V* while only one level (*E*_0_ = +*V*) is filled for a negative *V*. Figure 5f–h are for the cases with a finite mass, the Hall conductance is antisymmetric with respect to *V* and show very similar phase diagrams. Both odd and even integer quantum Hall phases can be induced by changing *V* in all the four cases. If setting *μ* = 0, we find that the Hall conductance is always vanishing, which is expected since the chemical potential *μ* is fixed at the center between the electron-like LLs that would go upward and the hole-like LLs that go downward when approaching the edge.

## Discussions

The formation of the edge states related to the two LLs near zero energy can be understood from the model that the top and bottom surfaces are separated by the metallic lateral surface. In the presence of a perpendicular magnetic field, the lateral surface electrons only experience an in-plane field, and the two Dirac points will shift oppositely by a constant if the Zeeman field is taken into account^30^. The edge states for the two LLs near the zero energy are divided into three parts: the parts of the wave function at the top and bottom surfaces decay exponentially away from the edge while they are connected by the part of the lateral surface electron. This is very similar to the case of the quantum anomalous Hall effect in a topological insulator thin film with a perpendicular Zeeman field^30^. A similar calculation can be found in a recent paper^50^. As the conductance of lateral surfaces is nonzero for a thin film with a finite thickness, the longitudinal conductance no longer vanishes. In this case the Hall resistance can be quantized perfectly only when the residual conductance from the lateral effect can be suppressed completely.

Mathematically the coupling between the top and bottom surfaces has been reasonably taken into account in the model in Eq. (1). Although it was shown rigorously that the Hall conductance for an ideal massless Dirac fermion gas with 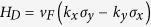 is quantized in a magnetic field as half integers, it is found that the Hall conductance is modified into integers once a quadratic correction 
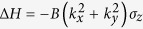
 is introduced to *H*_*D*_, where *σ*_*x*,*y*_,_*z*_ are the Pauli matrices. This is case (iii) in Fig. 2c. The conclusion is valid even if the parameter *B* is in the infinitesimal limit, and the solution of the edge states always exists. In other words, for an infinitesimal *B*, we obtain two well-defined edge states mathematically for two LLs of *n* = 0 as shown in Fig. 2e. The physical meaning of the term *B* is attributed to the coupling between the top and bottom surfaces according to the exact solution for a bulk model^39^,^40^.

Having in mind the picture of edge states of LLs for Dirac fermions we come to make some comments on the two experiments on the quantum Hall effect in topological insulator thin films. In the experiment by Xu *et al*.^37^, a series of the Hall conductance plateaux of *v* = −1, 0, 1, 2, 3 are measured as a function of the gate voltage *V*_*bg*_. The absence of the *v* = −2, −3 plateaux can be understood as the particle-hole symmetry breaking in the band structure of BiSbTeSe_2_, which is also confirmed by the transport measurement. The role of *V*_*bg*_ is to control the relative positions of the two Dirac points at the top and bottom surfaces, or the SIA term *V* in the thin film model in Eq. (1). The nonzero conductance *σ*_*xx*_ indicates that the Hall resistance has not yet been quantized completely. One of the reasons is that the thicknesses of the samples are 80 nm and 160 nm, respectively. In this case the lateral conductance does not vanish completely even at low temperatures. Suppression of the lateral effect will be a key to realize high precision of quantum Hall conductance in this experiment. On the odd integer quantum Hall effect, the plateaux of *v* = 1 and 3 are observed. As one surface of the thin film is grown on the substrate while the other is exposed to the vacuum, the boundary conditions are quite different, and the effective velocities of the surface electrons may not be identical. Thus it will be a hard task to make two sets of the LLs of the surface electrons degenerate completely by tuning the gate voltage only. However it is relatively easy to have only two specific LLs degenerate such that the plateau of *v* = 0 disappears.

In the experiment by Yoshimi *et al*.^38^, only two plateaux of *v* = −1, + 1 or *v* = 0, + 1 are measured in two different samples of (Bi_1−*x*_Sb_*x*_)_2_Te_3_. The presence of *v* = 0 plateau is attributed to the potential difference between the two surfaces, i.e., the SIA term 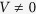. As the thickness of two samples is 8 nm, the finite-size effect is also relatively weak, i.e., 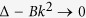. This should correspond to the case in Fig. 3. Thus the width of the plateau is determined by the value of *V*, and the pattern of edge states indicates that the state of *v* = 0 is simply a band insulator.

## Methods

### Landau levels

When a uniform field is applied perpendicular to the thin film, the wave vector is replaced by 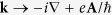, where the vector potential under the Landau gauge is 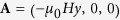. *k*_*x*_ remains a good quantum number. The LLs can be found by defining two ladder operators 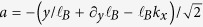 and 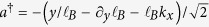, where the magnetic length 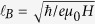, and assuming the trial solution,


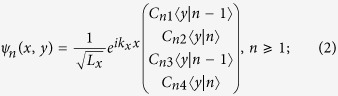



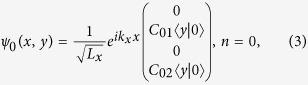


where 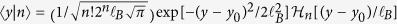 and 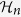 are Hermite polynomials. The energies of the LLs are found as


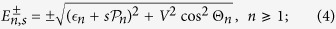



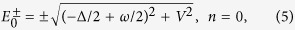


where 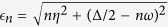, 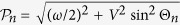, 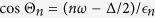, 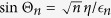, *s* = ±1, 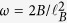, and 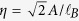. For each given 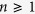, there are four LLs, two of them 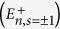 are electron-like while the other two 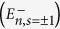 are hole-like. The expressions for the corresponding eigenstates can be found in the supplementary information.

The magnetic field can also induce a Zeeman energy described by 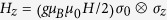 where *μ*_*B*_ is the Bohr magneton and *g* is the g-factor. It is weak, and thus we neglect it in this work. The Landau levels for the bulk states of a topological insulator was studied in a similar way^51^.

### Hall conductance

The Hall conductance can be found from the Kubo formula


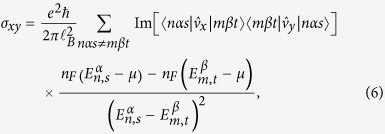


where 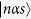 is the eigenstates in the LL 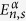, 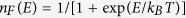 is the Fermi function with *k*_*B*_ the Boltzmann constant. 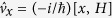 and 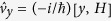 are two velocity operators.

### Edge states near open boundary

To solve the wave function of edge states at an open boundary, we employ the properties of the two standard solutions 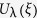 and 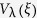 to the Weber equation^52^,


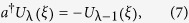



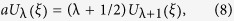



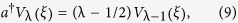



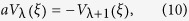


where the dimensionless quantity is defined by 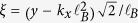. With the trial wave functions in the *y* direction


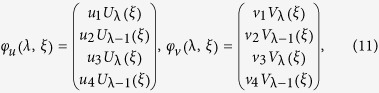


we can find the corresponding λ for a given eigen energy *E*. There are four λ’s for each given eigenenergy *E*, so a general solution to the wave function 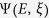 is a linear combination of eight eigenstates. The allowed eigen energies, the superposition coefficients as well as the wave function can be found by applying the open boundary conditions at the edge.

For a system with a semi-infinite geometry 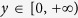, the wave function 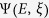 can only contain the 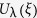 components since 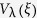 is exponentially divergent while 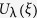 are vanishing as *ξ* approaches +∞ The boundary condition at *y* = 0 further provides an equation for *E*. In the weak coupling limit 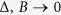, the equation for *E* is reduced as


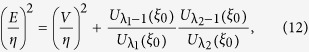


where 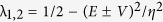 and 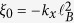. If *E* is a solution, then −*E* is also a solution, reflecting the particle-hole symmetry, as expected. Thus we shows that the solutions of edge states exist even if 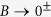.

## Additional Information

**How to cite this article**: Zhang, S.-B. *et al*. Edge states and integer quantum Hall effect in topological insulator thin films. *Sci. Rep*. **5**, 13277; doi: 10.1038/srep13277 (2015).

## Supplementary Material

Supplementary Information

## References

[b1] Klitzing K. V., Dorda G. & Pepper M. New method for high-accuracy determination of the fine-structure constant based on quantized Hall resistance. Phys. Rev. Lett. 45, 494–497 (1980).

[b2] Prange R. E., Girvin S. M. & V. Klitzing K. The quantum Hall effect (Springer, 1989).

[b3] Thouless D. J., Kohmoto M., Nightingale M. P. & Den Nijs M. Quantized Hall conductance in a two-dimensional periodic potential. Phys. Rev. Lett. 49, 405–408 (1982).

[b4] Halperin B. I. Quantized Hall conductance, current-carrying edge states, and the existence of extended states in a two-dimensional disordered potential. Phys. Rev. B 25, 2185–2190 (1982).

[b5] MacDonald A. H. & Středa P. Quantized Hall effect and edge currents. Phys. Rev. B 29, 1616–1619 (1984).

[b6] Hatsugai Y. Chern number and edge states in the integer quantum Hall effect. Phys. Rev. Lett. 71, 3697–3700 (1993). 10.1103/PhysRevLett.71.3697 10055049

[b7] Moore J. E. The birth of topological insulators. Nature 464, 194–198 (2010). 10.1038/nature08916 20220837

[b8] Hasan M. Z. & Kane C. L. *Colloquium* : Topological insulators. Rev. Mod. Phys. 82, 3045–3067 (2010).

[b9] Qi X.-L. & Zhang S.-C. Topological insulators and superconductors. Rev. Mod. Phys. 83, 1057–1110 (2011).

[b10] Shen S.-Q. Topological Insulators (Springer, 2012).

[b11] Hanaguri T., Igarashi K., Kawamura M., Takagi H. & Sasagawa T. Momentum-resolved Landau-level spectroscopy of Dirac surface state in Bi_2_Se_3_. Phys. Rev. B 82, 081305 (2010).

[b12] Cheng P. . Landau quantization of topological surface states in Bi_2_Se_3_. Phys. Rev. Lett. 105, 076801 (2010). 20868065 10.1103/PhysRevLett.105.076801

[b13] Fu Y.-S. . Imaging the two-component nature of Dirac-Landau levels in the topological surface state of Bi_2_Se_3_. Nat. Phys. 10, 815–819 (2014).

[b14] Qu D.-X., Hor Y. S., Xiong J., Cava R. J. & Ong N. P. Quantum oscillations and Hall anomaly of surface states in the topological insulator Bi_2_Te_3_. Science 329, 821–824 (2010). 20671155 10.1126/science.1189792

[b15] Analytis J. G. . Two-dimensional surface state in the quantum limit of a topological insulator. Nat. Phys. 6, 960–964 (2010).

[b16] Brüne C. . Quantum Hall effect from the topological surface states of strained bulk HgTe. Phys. Rev. Lett. 106, 126803 (2011). 21517339 10.1103/PhysRevLett.106.126803

[b17] Jackiw R. Fractional charge and zero modes for planar systems in a magnetic field. Phys. Rev. D 29, 2375–2377 (1984). 10.1103/physrevd.33.2500 9956933

[b18] Schakel A. M. J. Relativistic quantum Hall effect. Phys. Rev. D 43, 1428–1431 (1991). 10.1103/physrevd.43.1428 10013517

[b19] Ando T., Nakanishi T. & Saito R. Berry’s phase and absence of back scattering in carbon nanotubes. Journal of the Physical Society of Japan 67, 2857–2862 (1998).

[b20] Gusynin V. P. & Sharapov S. G. Unconventional integer quantum Hall effect in graphene. Phys. Rev. Lett. 95, 146801 (2005). 16241680 10.1103/PhysRevLett.95.146801

[b21] Lee D.-H. Surface states of topological insulators: The Dirac fermion in curved two-dimensional spaces. Phys. Rev. Lett. 103, 196804 (2009). 20365943 10.1103/PhysRevLett.103.196804

[b22] Mikitik G. P. & Sharlai Y. V. Manifestation of Berry’s phase in metal physics. Phys. Rev. Lett. 82, 2147–2150 (1999).

[b23] Luk’yanchuk I. A. & Kopelevich Y. Phase analysis of quantum oscillations in graphite. Phys. Rev. Lett. 93, 166402 (2004). 15525015 10.1103/PhysRevLett.93.166402

[b24] Abanin D. A., Lee P. A. & Levitov L. S. Spin-filtered edge states and quantum Hall effect in graphene. Phys. Rev. Lett. 96, 176803 (2006). 16712323 10.1103/PhysRevLett.96.176803

[b25] Peres N. M. R., Guinea F. & Castro Neto A. H. Electronic properties of disordered two-dimensional carbon. Phys. Rev. B 73, 125411 (2006).

[b26] Zhou B., Ren L. & Shen S.-Q. Spin transverse force and intrinsic quantum transverse transport. Phys. Rev. B 73, 165303 (2006).

[b27] Qi X.-L., Hughes T. L. & Zhang S.-C. Topological field theory of time-reversal invariant insulators. Phys. Rev. B 78, 195424 (2008).

[b28] Qi X.-L., Li R., Zang J. & Zhang S.-C. Inducing a magnetic monopole with topological surface states. Science 323, 1184–1187 (2009). 19179491 10.1126/science.1167747

[b29] Tkachov G. & Hankiewicz E. M. Ballistic quantum spin Hall state and enhanced edge backscattering in strong magnetic fields. Phys. Rev. Lett. 104, 166803 (2010). 20482073 10.1103/PhysRevLett.104.166803

[b30] Chu R.-L., Shi J. & Shen S.-Q. Surface edge state and half-quantized Hall conductance in topological insulators. Phys. Rev. B 84, 085312 (2011).

[b31] Vafek O. Quantum Hall effect in a singly and doubly connected three-dimensional topological insulator. Phys. Rev. B 84, 245417 (2011).

[b32] Zyuzin A. A. & Burkov A. A. Thin topological insulator film in a perpendicular magnetic field. Phys. Rev. B 83, 195413 (2011).

[b33] Sitte M., Rosch A., Altman E. & Fritz L. Topological insulators in magnetic fields: Quantum Hall effect and edge channels with a nonquantized θ term. Phys. Rev. Lett. 108, 126807 (2012). 22540614 10.1103/PhysRevLett.108.126807

[b34] Zhang Y.-Y., Wang X.-R. & Xie X. C. Three-dimensional topological insulator in a magnetic field: chiral side surface states and quantized Hall conductance. J. Phys.: Condens. Matter 24, 015004 (2012). 22134101 10.1088/0953-8984/24/1/015004

[b35] Li G., Luican-Mayer A., Abanin D., Levitov L. & Andrei E. Y. Evolution of Landau levels into edge states in graphene. Nat. Commun. 4, 1744 (2013). 23612285 10.1038/ncomms2767

[b36] König E. J. . Half-integer quantum Hall effect of disordered Dirac fermions at a topological insulator surface. Phys. Rev. B 90, 165435 (2014).

[b37] Xu Y. . Observation of topological surface state quantum Hall effect in an intrinsic three-dimensional topological insulator. Nat. Phys. 10, 956–963 (2014).

[b38] Yoshimi R. . Quantum Hall effect on top and bottom surface states of topological insulator (Bi_1−x_Sb_x_)_2_Te_3_ films. ArXiv e-prints 1409.3326 (2014). 10.1038/ncomms7627 25868494

[b39] Lu H.-Z., Shan W.-Y., Yao W., Niu Q. & Shen S.-Q. Massive Dirac fermions and spin physics in an ultrathin film of topological insulator. Phys. Rev. B 81, 115407 (2010).

[b40] Shan W.-Y., Lu H.-Z. & Shen S.-Q. Effective continuous model for surface states and thin films of three-dimensional topological insulators. New J. Phys. 12, 043048 (2010).

[b41] Zhang H. . Topological insulators in Bi_2_Se_3_, Bi_2_Te_3_ and Sb_2_Te_3_ with a single Dirac cone on the surface. Nat. Phys. 5, 438–442 (2009).

[b42] Zhang Y. . Crossover of the three-dimensional topological insulator Bi_2_Se_3_ to the two-dimensional limit. Nat. Phys. 6, 584–588 (2010).

[b43] Sakamoto Y., Hirahara T., Miyazaki H., Kimura S.-i. & Hasegawa S. Spectroscopic evidence of a topological quantum phase transition in ultrathin Bi_2_Se_3_ films. Phys. Rev. B 81, 165432 (2010).

[b44] Novoselov K. S. . Two-dimensional gas of massless Dirac fermions in graphene. Nature 438, 197–200 (2005). 16281030 10.1038/nature04233

[b45] Zhang Y., Tan Y.-W., Stormer H. L. & Kim P. Experimental observation of the quantum Hall effect and Berry’s phase in graphene. Nature 438, 201–204 (2005). 16281031 10.1038/nature04235

[b46] König M. . Quantum spin Hall insulator state in HgTe quantum wells. Science 318, 766–770 (2007). 17885096 10.1126/science.1148047

[b47] Kane C. L. & Mele E. J. Quantum spin Hall effect in graphene. Phys. Rev. Lett. 95, 226801 (2005). 16384250 10.1103/PhysRevLett.95.226801

[b48] Bernevig B. A., Hughes T. L. & Zhang S.-C. Quantum spin Hall effect and topological phase transition in HgTe quantum wells. Science 314, 1757–1761 (2006). 17170299 10.1126/science.1133734

[b49] Zhang S.-B., Zhang Y.-Y. & Shen S.-Q. Robustness of quantum spin Hall effect in an external magnetic field. Phys. Rev. B 90, 115305 (2014).

[b50] Morimoto T., Furusaki A. & Nagaosa N. Charge and spin transport in edge channels of a ν = 0 quantum Hall system on the surface of topological insulators. ArXiv e-prints 1412.8359 (2014). 10.1103/PhysRevLett.114.146803 25910149

[b51] Yang Z. & Han J. H. Landau level states on a topological insulator thin film. Phys. Rev. B 83, 045415 (2011).

[b52] Abramowitz M. & Stegun I. A. Handbook of mathematical functions with formulas, graphs, and mathematical tables (U.S. government printing office, Washington, D.C., 1972).

