# Supplementary material for: Edge states and integer quantum Hall effect in topological insulator thin films
Source: Sci Rep. 2015 Aug 25;5:13277. doi: 10.1038/srep13277 (PMC4548229; doi:10.1038/srep13277)
Supplement: Supplementary Information [file srep13277-s1.pdf]

Supplementary information
for
"Edge states and integer quantum Hall effect in topological insulator thin films"

Song-Bo Zhang, Hai-Zhou Lu, & Shun-Qing Shen
Department of Physics, The University of Hong Kong, Pokfulam Road, Hong Kong, China

SUPPLEMENTARY NOTE 1 | SOLUTIONS OF LANDAU LEVELS.

As introduced in the main text, we use the Landau gauge $\mathbf{A} = (-\mu_0 H y, 0, 0)$ and define two ladder operators a and $a^\dagger$, then we can write the Hamiltonian as

$$H = \begin{pmatrix} M_a & i\eta a & V & 0 \\ -i\eta a^\dagger & -M_a & 0 & V \\ V & 0 & -M_a & i\eta a \\ 0 & V & -i\eta a^\dagger & M_a \end{pmatrix}, \tag{1}$$

where $M_a = \Delta/2 - \omega(a^\dagger a + 1/2)$, $\omega = 2B/\ell_B^2$, and $\eta = \sqrt{2}\gamma/\ell_B$. Using the trial solution given by Eq. (2) in the main text and solving the eigen equations, we find that energies of the Landau levels (LLs) are given by

$$E_{n,s}^{\pm} = \pm\sqrt{(\epsilon_n + s\mathcal{P}_n)^2 + V^2\cos^2\Theta_n}, \quad n \geqslant 1; \tag{2}$$

$$E_0^{\pm} = \pm\sqrt{(-\Delta/2 + \omega/2)^2 + V^2}, \quad n = 0 \tag{3}$$

where $\epsilon_n = \sqrt{n\eta^2 + (\Delta/2 - n\omega)^2}$, $\mathcal{P}_n = \sqrt{(\omega/2)^2 + V^2\sin^2\Theta_n}$, $\cos\Theta_n = (n\omega - \Delta/2)/\epsilon_n$, $\sin\Theta_n = \sqrt{n}\eta/\epsilon_n$, and $s = \pm 1$. The corresponding LL eigenstates $|n\alpha s\rangle$ $(n \geqslant 1)$ are explicitly given by

$$|n+, s=+1\rangle = \begin{pmatrix} \mathcal{F}_{sc-}^{+}|n-1\rangle \\ \mathcal{F}_{ss+}^{-}|n\rangle \\ -i\mathcal{F}_{cc-}^{-}|n-1\rangle \\ -i\mathcal{F}_{cs+}^{+}|n-1\rangle \end{pmatrix}, \qquad |n+, s=-1\rangle = \begin{pmatrix} -i\mathcal{F}_{cc+}^{+}|n-1\rangle \\ -i\mathcal{F}_{cs-}^{-}|n\rangle \\ \mathcal{F}_{sc+}^{+}|n-1\rangle \\ \mathcal{F}_{ss-}^{+}|n\rangle \end{pmatrix}, \tag{4}$$

$$|n-, s=+1\rangle = \begin{pmatrix} -i\mathcal{F}_{ss-}^{+}|n-1\rangle \\ i\mathcal{F}_{sc+}^{-}|n\rangle \\ \mathcal{F}_{cs-}^{-}|n-1\rangle \\ -\mathcal{F}_{cc+}^{+}|n\rangle \end{pmatrix}, \qquad |n-, s=-1\rangle \begin{pmatrix} \mathcal{F}_{cs+}^{+}|n-1\rangle \\ -\mathcal{F}_{cc-}^{-}|n\rangle \\ -i\mathcal{F}_{ss+}^{-}|n-1\rangle \\ i\mathcal{F}_{sc-}^{+}|n\rangle \end{pmatrix}, \tag{5}$$

where

$$\mathcal{F}_{ss,\pm}^{p} = \sin\frac{\Theta_n}{2}\sin\frac{\phi_n}{2}\cos\frac{\varphi_n^p}{2} \pm \cos\frac{\Theta_n}{2}\cos\frac{\phi_n}{2}\sin\frac{\varphi_n^p}{2}, \tag{6}$$

$$\mathcal{F}_{sc,\pm}^{p} = \sin\frac{\Theta_n}{2}\cos\frac{\phi_n}{2}\cos\frac{\varphi_n^p}{2} \pm \cos\frac{\Theta_n}{2}\sin\frac{\phi_n}{2}\sin\frac{\varphi_n^p}{2}, \tag{7}$$

$$\mathcal{F}_{cs,\pm}^{p} = \cos\frac{\Theta_n}{2}\sin\frac{\phi_n}{2}\cos\frac{\varphi_n^p}{2} \pm \sin\frac{\Theta_n}{2}\cos\frac{\phi_n}{2}\sin\frac{\varphi_n^p}{2}, \tag{8}$$

$$\mathcal{F}_{cc,\pm}^{p} = \cos\frac{\Theta_n}{2}\cos\frac{\phi_n}{2}\cos\frac{\varphi_n^p}{2} \pm \sin\frac{\Theta_n}{2}\sin\frac{\phi_n}{2}\sin\frac{\varphi_n^p}{2}. \tag{9}$$

with $\cos\phi_n = \omega/(2\mathcal{P}_n)$, $\sin\phi_n = V\sin\Theta_n/\mathcal{P}_n$, $\cos\varphi_n^{\pm} = (\epsilon_n \pm \mathcal{P}_n)/|E_{n,\pm 1}^{+}|$, and $\sin\varphi_n^{\pm} = V\cos\Theta_n/|E_{n,\pm 1}^{+}|$. The eigenstates for the two $n = 0$ LLs are

$$|0,+\rangle = \begin{pmatrix} 0 \\ \sin\frac{\Gamma}{2}|0\rangle \\ 0 \\ \cos\frac{\Gamma}{2}|0\rangle \end{pmatrix}, \quad |0,-\rangle = \begin{pmatrix} 0 \\ \cos\frac{\Gamma}{2}|0\rangle \\ 0 \\ -\sin\frac{\Gamma}{2}|0\rangle \end{pmatrix}, \tag{10}$$

where $\cos\Gamma = (\Delta - \omega)/(2|E_0^{+}|)$ and $\sin\Gamma = V/|E_0^{+}|$.

SUPPLEMENTARY NOTE 2 | CALCULATION OF HALL CONDUCTANCE.

The velocity operators $\hat{v}_x$ and $\hat{v}_y$ in the form of matrix are given by

$$\hat{v}_x = -\frac{\ell_B}{\sqrt{2}\hbar}\begin{pmatrix} \omega A_+ & -i\eta & 0 & 0 \\ i\eta & -\omega A_+ & 0 & 0 \\ 0 & 0 & -\omega A_+ & -i\eta \\ 0 & 0 & i\eta & \omega A^+ \end{pmatrix}, \tag{11}$$

and

$$\hat{v}_y = -i\frac{\ell_B}{\sqrt{2}\hbar}\begin{pmatrix} -\omega A_- & i\eta & 0 & 0 \\ i\eta & \omega A_- & 0 & 0 \\ 0 & 0 & \omega A_- & i\eta \\ 0 & 0 & i\eta & -\omega A_- \end{pmatrix}, \tag{12}$$

where $A_\pm = a^\dagger \pm a$. Substituting the velocity operators, the eigen energies Eqs. (2-3) and eigenstates Eqs. (4-10) into the Kubo formula, we can obtain

$$\begin{aligned}\sigma_{xy} = -\frac{e^2}{h}\Bigg[&\sum_{\alpha,\beta,t}\left|\xi^{0\alpha}_{1\beta t}\right|^2\frac{n_F(E^\alpha_0-\mu)-n_F(E^\beta_{1,t}-\mu)}{(E^\alpha_0-E^\beta_{1,t})^2} \\ &+\sum_{n\geqslant 1}\sum_{\alpha,\beta,s,t}\left|f^{n\alpha s}_{n+1\beta t}+g^{n\alpha s}_{n+1\beta t}\right|^2\frac{n_F(E^\alpha_{n,s}-\mu)-n_F(E^\beta_{n+1,t}-\mu)}{(E^\alpha_{n,s}-E^\beta_{n+1,t})^2}\Bigg],\end{aligned} \tag{13}$$

where

$$f^{n\alpha s}_{m\beta t} = \omega\Big(C^{\alpha s*}_{n1}C^{\beta t}_{m1}\sqrt{n} - C^{\alpha s*}_{n3}C^{\beta t}_{m3}\sqrt{n} - C^{\alpha s*}_{n2}C^{\beta t}_{m2}\sqrt{m} + C^{\alpha s*}_{n4}C^{\beta t}_{m4}\sqrt{m}\Big), \tag{14}$$

$$g^{n\alpha s}_{m\beta t} = i\eta\Big(C^{\alpha s*}_{n2}C^{\beta t}_{m1} + C^{\alpha s*}_{n4}C^{\beta t}_{m3}\Big), \tag{15}$$

$$\xi^{0\alpha}_{1\beta t} = \omega\Big(C^{\alpha*}_{02}C^{\beta t}_{14} - C^{\alpha*}_{01}C^{\beta t}_{12}\Big) + i\eta\Big(C^{\alpha*}_{01}C^{\beta t}_{11} + C^{\alpha*}_{02}C^{\beta t}_{13}\Big). \tag{16}$$

$C^{\alpha s}_{ni}$ is the coefficient of the i-th component in the eigenstate $|n\alpha s\rangle$ Eq. (4-10), and is explicitly given by the $\mathcal{F}$ function (6-9).

SUPPLEMENTARY NOTE 3 | EDGE STATES AT OPEN BOUNDARY.

Without SIA

In the absence of SIA, i.e., $V = 0$, the Hamiltonian (1) is block-diagonalized and the Hamiltonian for one block is given by

$$h_1(\Delta, B) = \begin{pmatrix} M_a & i\eta a \\ -i\eta a^\dagger & -M_a \end{pmatrix}, \tag{17}$$

and the Hamiltonian $h_2(\Delta, B)$ for the other block is related to h_1 by $h_2(\Delta, B) = h_1(-\Delta, -B)$. Using the trial wave function,

$$\phi_u(\lambda, \xi) = \begin{pmatrix} u_1 U_\lambda(\xi) \\ u_2 U_{\lambda-1}(\xi) \end{pmatrix}, \quad \phi_v(\lambda, \xi) = \begin{pmatrix} v_1 V_\lambda(\xi) \\ v_2 V_{\lambda-1}(\xi) \end{pmatrix}, \tag{18}$$

we find two λ's for each given eigenenergy E

$$\lambda_\pm = \frac{\mathcal{K} \pm \sqrt{\mathcal{K}^2 + 4\omega^2\mathcal{M}}}{2\omega^2}, \tag{19}$$

where

$$\mathcal{K} = \eta^2 + \omega^2 - \Delta\omega, \tag{20}$$

$$\mathcal{M} = -\frac{\eta^2}{2} + (E - \frac{\Delta}{2})(E + \frac{\Delta}{2} - \omega). \tag{21}$$

Each λ corresponds to two linearly independent eigenstates of the system without boundary

$$\phi_u(\lambda_\pm, \xi) = \begin{pmatrix} [\Delta/2 + E + \omega(\lambda_\pm - 1)]U_{\lambda_\pm}(\xi) \\ i\eta U_{\lambda_\pm - 1}(\xi) \end{pmatrix}, \tag{22}$$

and

$$\phi_v(\lambda_\pm, \xi) = \begin{pmatrix} i\eta V_{\lambda_\pm}(\xi) \\ (\Delta/2 - E + \omega\lambda_\pm)V_{\lambda_\pm - 1}(\xi) \end{pmatrix}. \tag{23}$$

A general wave function is then a linear combination of four eigenstates

$$\Phi(\xi, E) = \sum_{\gamma=\pm} \Big[C^u_\gamma \phi_u(\lambda_\gamma, \xi) + C^v_\gamma \phi_v(\lambda_\gamma, \xi) \Big]. \tag{24}$$

Consider a semi-infinite geometry $y \in [0, +\infty)$ with open boundary conditions. Since $V_\lambda(\xi)$ are exponentially divergent while $U_\lambda(\xi)$ are vanishing as ξ approaches $+\infty$, the wave function $\Psi(\xi, E)$ can only contain the $U_\lambda(\xi)$ components as required by the normalizablity. The boundary condition $\Psi(\xi_0, E) = 0$ with $\xi_0 \equiv -\sqrt{2}k_x\ell_B$ then gives an equation to determine the eigenenergies E for the semi-infinite system

$$\frac{[E + \omega(\lambda_- - 1)]}{[E + \omega(\lambda_+ - 1)]} \frac{U_{\lambda_-}(\xi_0)}{U_{\lambda_+}(\xi_0)} \frac{U_{\lambda_+ - 1}(\xi_0)}{U_{\lambda_- - 1}(\xi_0)} = 1. \tag{25}$$

Solving this equation of E for each given k_x, we obtain the energy dispersions of LLs with an open boundary.

In the limit $\Delta, B \to 0$, the two λ are approximated by

$$\lambda_- \simeq \frac{1}{2} - \frac{E^2}{\eta^2}, \quad \lambda_+ \simeq \frac{\eta^2}{\omega^2} \to \infty. \tag{26}$$

As $\xi_0^2 \ll \lambda_+$, we can use [1]

$$U_{\lambda_+}(\xi_0) \sim \frac{\sqrt{\pi}}{2^{\frac{\lambda_+}{2} + \frac{1}{4}} \Gamma(\frac{3}{4} + \frac{\lambda_+}{2})} e^{-\sqrt{\lambda_+}\xi_0} \tag{27}$$

where $\Gamma(t)$ is the Gamma function. Using the asymptotic expression for the $\Gamma(t)$ function when $t \to \infty$,

$$\Gamma(t+1) \sim \sqrt{2\pi t}(\frac{t}{e})^t, \tag{28}$$

where e is Euler's number which can be expressed as

$$\frac{1}{e} = \lim_{x\to\infty} (1 + \frac{1}{x})^x, \tag{29}$$

we find that

$$\frac{U_{\lambda_+ - 1}(\xi_0)}{U_{\lambda_+}(\xi_0)} \sim \sqrt{\lambda_+} \sim \Big|\frac{\eta}{\omega}\Big|. \tag{30}$$

On the other hand, we have

$$\frac{[E + \omega(\lambda_- - 1)]}{[E + \omega(\lambda_+ - 1)]} \sim \frac{E\omega}{\eta^2}. \tag{31}$$

Therefore, we can finally reduce Eq. (25) as

$$\frac{E}{|\eta|} = \mathrm{sgn}(B) \frac{U_{\lambda_- - 1}(\xi_0)}{U_{\lambda_-}(\xi_0)}. \tag{32}$$

We can use this equation to determine the energy dispersions of LLs in the limit $\Delta, B \to 0$, while use Eq. (25) to determine the edge dispersions for a finite B case. Supplementary Figure 1 shows the edge dispersion of the LL of $n = 0$ for several values of B near the edge. The LL of $n = 0$ suddenly changes from electron-like to hole-like when B changes from a positive infinitesimal to a negative infinitesimal.

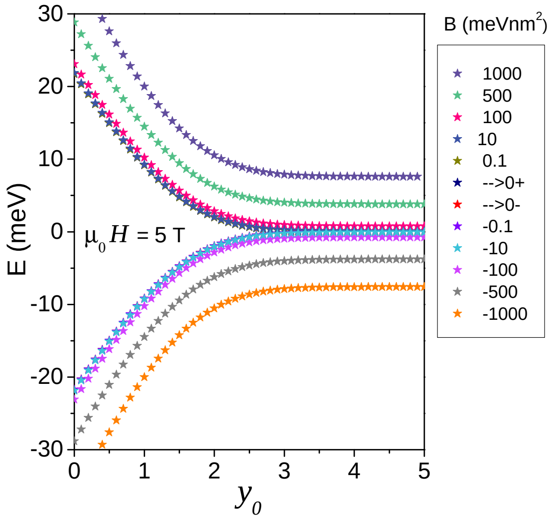

Supplementary Figure 1 | Edge states of the $n = 0$ LL of h_1 for different values of B. When B is varied from 1000 to 0^+ meVnm^2, the energy dispersion near the edge converges into an electron-like band. Then it suddenly changes from electron-like to hole-like when B changes from a positive infinitesimal to a negative infinitesimal, and finally evolutes away as a hole-like band. The other parameters are $\gamma = 300$ meVnm and $\Delta = 0$.

With SIA

In the presence of SIA, we shall put the trial wave functions in terms of $U_\lambda(\xi)$ and $V_\lambda(\xi)$ functions Eq. (10) in the main text into the eigen equation, and then find four λ's for each given E. The general wave function $\Psi(E, \xi)$ is then constructed as a linear combination of eight eigenstates.

$$\Psi(\xi, E) = \sum_{i=1}^{4} \Big[C_i^u \varphi_u(\lambda_i, \xi) + C_i^v \varphi_v(\lambda_i, \xi) \Big]. \tag{33}$$

The allowed eigenenergies E and the superposition coefficients are found by applying the open boundary condition at the boundary.

For the semi-infinite geometry $y \in [0, +\infty)$ with open boundary conditions, the wave function $\Psi(\xi, E)$ can only contain the $U_\lambda(\xi)$ components. The boundary condition $\Psi(\xi_0 =, E) = 0$ can be rewritten as a determinant of a 4×4 matrix

$$\big| \varphi_u(\lambda_1, \xi_0) \;\; \varphi_u(\lambda_2, \xi_0) \;\; \varphi_u(\lambda_3, \xi_0) \;\; \varphi_u(\lambda_4, \xi_0) \big| = 0. \tag{34}$$

The solutions of E for each given k_x form the edge dispersions of LLs.

Generally speaking, in the four λ's, $\lambda_{1,2}$ are real while the other two $\lambda_{3,4}$ are complex and $\lambda_3 = \lambda_4^*$. The explicit expressions for the four λ's and the four corresponding eigenstates $\varphi_u(\lambda_i, \xi_0)$ $(i = 1, 2, 3, 4)$ can be found, but are too complex. However, in the small coupling limit $\Delta, B \to 0$, we have the two real λ's as $\lambda_{1,2} = 1/2 - (V \pm E)^2/\eta^2$ and the corresponding eigenstates

$$\varphi_u(\lambda_\rho, E) = \begin{pmatrix} i\dfrac{V + (-1)^{\rho-1}E}{\eta} U_{\lambda_\rho}(\xi) \\ (-1)^\rho U_{\lambda_\rho - 1}(\xi) \\ i\dfrac{(-1)^\rho V - E}{\eta} U_{\lambda_\rho}(\xi) \\ -U_{\lambda_\rho - 1}(\xi) \end{pmatrix}, \qquad \rho = 1, 2. \tag{35}$$

The two complex λ's can be approximated by $\lambda_{3,4} \sim \eta^2/\omega^2 \pm i\delta$ with $|\delta| \ll |\lambda_{3,4}|$ and the corresponding eigenstates

are

$$\varphi_u(\lambda_\tau, E) = \begin{pmatrix} i\frac{2V^2 - 2E^2 - \eta^2 + (-1)^{\tau-1}2i\delta\eta^2}{4V\eta}U_{\lambda_\tau}(\xi) \\ \omega\frac{2V^2 + 2E^2 + \eta^2 + (-1)^{\tau}2i\delta\eta^2}{4V\eta^2}U_{\lambda_\tau - 1}(\xi) \\ i\frac{\eta}{\omega}U_{\lambda_\tau}(\xi) \\ -U_{\lambda_\tau}(\xi) \end{pmatrix}, \quad \tau = 3, 4. \tag{36}$$

Putting Eqs. (35-36) into the boundary condition Eq. (34) and using the following approximation at $\omega \to 0$

$$\frac{U_{\lambda_\tau - 1}(\xi_0)}{U_{\lambda_\tau}(\xi_0)} \sim \sqrt{\lambda_3} = \left|\frac{\eta}{\omega}\right| + i\epsilon, \quad \tau = 3, 4, \tag{37}$$

where $|\epsilon| \ll |\eta/\omega|$ and $|\delta/(2\epsilon)| \sim |\eta/\omega| \gg 1$, the equation of E is finally simplified as

$$\left(\frac{E}{\eta}\right)^2 = \left(\frac{V}{\eta}\right)^2 + \frac{U_{\lambda_1 - 1}(\xi_0)}{U_{\lambda_1}(\xi_0)}\frac{U_{\lambda_2 - 1}(\xi_0)}{U_{\lambda_2}(\xi_0)}. \tag{38}$$

This is the equation that we can use to find the energy dispersion for the massless surface electrons near the edge.

When $V = 0$, $\lambda_1 = \lambda_2 = \lambda \equiv 1/2 - E^2/\eta^2$ and thus Eq. (38) is further reduced to

$$\frac{E}{\eta} = \pm\frac{U_{\lambda-1}(\xi_0)}{U_\lambda(\xi_0)}. \tag{39}$$

We can see that one of these two equation must resemble to Eq. (32).

[1] Abramowitz, M. & Stegun, I. A. *Handbook of mathematical functions with formulas, graphs, and mathematical tables* (U.S. government printing office, Washington, D.C., 1972).
